# Supplementary material for: Data-Independent Acquisition Mass Spectrometry as a Tool for Metaproteomics: Interlaboratory Comparison Using a Model Microbiome
Source: bioRxiv. 2025 Jan 2:2024.09.18.613707. Originally published 2024 Sep 22. Preprint. [Version 2] doi: 10.1101/2024.09.18.613707 (PMC11430069; doi:10.1101/2024.09.18.613707)

Supplemental Information for Data-Independent Acquisition Mass Spectrometry as a Tool for Metaproteomics: Cross-Laboratory Methodological Comparisons Using a Model Microbiome

Rajczewski, A. T.[1], Blakeley-Ruiz, J. A.[2], Meyer, A.[3], Vintila, S.[2], McIlvin, M.R.[4], Van Den Bossche, T.[5, 6], Searle, B.C.[7], Griffin, T.J.[1], Saito, M.[4], Kleiner, M.[2], Jagtap, P.D.[1]

[1] Department of Biochemistry, Molecular Biology, and Biophysics, University of Minnesota, Minneapolis MN USA
[2] Department of Plant and Microbial Biology, North Carolina State University, Raleigh NC USA
[3] MIT-WHOI Joint Program in Oceanography/Applied Ocean Science and Engineering, Department of Chemistry, Woods Hole Oceanographic Institution, Woods Hole MA USA, Department of Earth, Atmospheric, and Planetary Sciences, Massachusetts Institute of Technology, Cambridge MA USA
[4] Department of Marine Chemistry and Geochemistry, Woods Hole Oceanographic Institution, Woods Hole MA USA
[5] VIB-UGent Center for Medical Biotechnology, VIB,, Ghent Belgium
[6] Department of Biomolecular Medicine, Ghent University, Ghent Belgium
[7] Department of Chemistry and Biochemistry, The Ohio State University, Columbus OH USA

Supplemental Table S1: Protein sequence database components used in the proteomic analyses of composite microbiome samples

**Label**	**Species**	Source of protein sequences	Original Database	Incomplete 1	Incomplete 2	Added1	Added Incomplete1	Added Incomplete2
NV	*Nitrososphaera viennensis*	GCA_000698785.1	Y	Y	Y	Y	Y	Y
841	*Rhizobium leguminosarum bv. viciae* 3841	UP000006575	Y	N	N	Y	N	N
PaD	*Paracoccus denitrificans*	Used RAST. Available in the protein sequence database on PRIDE.	Y	Y	Y	Y	Y	Y
Cup	*Cupriavidus metallidurans*	UP000002429	Y	Y	Y	Y	Y	Y
CV	*Chromobacterium violaceum*	Used RAST. Available in the protein sequence database on PRIDE.	Y	Y	Y	Y	Y	Y
Nu1	*Nitrosomonas ureae*	UP000056699	Y	Y	Y	Y	Y	Y
DVH	*Desulfovibrio vulgaris*	UP000002194	Y	Y	Y	Y	Y	Y
K12	*Escherichia coli*	UP000000625	Y	Y	Y	Y	Y	Y
PD	*Pseudomonas denitrificans*	UP000012082	Y	N	N	Y	N	N
KF7	*Pseudomonas pseudoalcaligenes*	GCA_000262065.3	Y	Y	N	Y	Y	N
Pfl	*Pseudomonas fluorescens*	2617270901 (IMG)	Y	N	N	Y	N	N
HB2	*Thermus Thermophilus*	UP000000592	Y	Y	Y	Y	Y	Y
CRH	*Chlamydomonas reinhardtii*	GCF_000002595.1	Y	Y	Y	Y	Y	Y
LT2	*Salmonella enterica Typhimurium* (3 strains combined)	UP000001014	Y	Y	N	Y	Y	N

ATN	*Agrobacterium tumefaciens*	UP00000813	Y	Y	Y	Y	Y	Y
VF	*Rhizobium leguminosarum bv. viciae* VF39	GCA_000427765.1	Y	Y	N	Y	Y	N
AK199	*Roseobacter sp.* AK199	Used RAST. Available in the protein sequence database on PRIDE.	Y	Y	Y	Y	Y	Y
BXL	*Burkholderia xenovorans*	UP000001817	Y	Y	Y	Y	Y	Y
Ne1	*Nitrosomonas europaeae*	UP000001416	Y	Y	Y	Y	Y	Y
Nm1	*Nitrosospira multiformis*	UP000002718	Y	Y	Y	Y	Y	Y
Am2	*Alteromonas macleodii*	UP000006296	Y	Y	Y	Y	Y	Y
SMS	*Stenotrophomonas maltophilia*	GCF_000613205.1	Y	Y	Y	Y	Y	Y
BS	*Bacillus subtilis*	UP000001570	Y	Y	Y	Y	Y	Y
137	*Staphylococcus aureus* ATCC 13709	Used RAST.. Available in the protein sequence database on PRIDE	Y	Y	Y	Y	Y	Y
259	*Staphylococcus aureus* ATCC 25923	GCA_000756205.1	Y	Y	Y	Y	Y	Y
ES18	Phage ES18	UP000000970	Y	Y	Y	Y	Y	Y
F0	Phage F0	UP000009070	Y	Y	Y	Y	Y	Y
F2	Phage F2	UP000002127	Y	Y	Y	Y	Y	Y
M13	Phage M13	UP000002111	Y	Y	Y	Y	Y	Y
P22	Phage P22	UP000007960	Y	Y	Y	Y	Y	Y
BT	*Bacteroides thetaiotaomicron*	UP00000141	N	N	N	Y	Y	Y
BBE	*Buttiauxella brennerae*	UP00007841	N	N	N	Y	Y	Y
SBI	*Salmonella bongori*	UP00027208	N	N	N	Y	Y	Y
TMO	*Tistrella mobilis*	UP00000525	N	N	N	Y	Y	Y

Supplemental Figure S1: UpSet plots of peptide reproducibility of Equal Cell Number (red) and Equal Protein Amount (green) communities across three mass spectrometers (QExactive, Fusion, and Eclipse) under DDA- and DIA-MS analysis. The variables n1 through n4 represent MS runs of the four replicates.

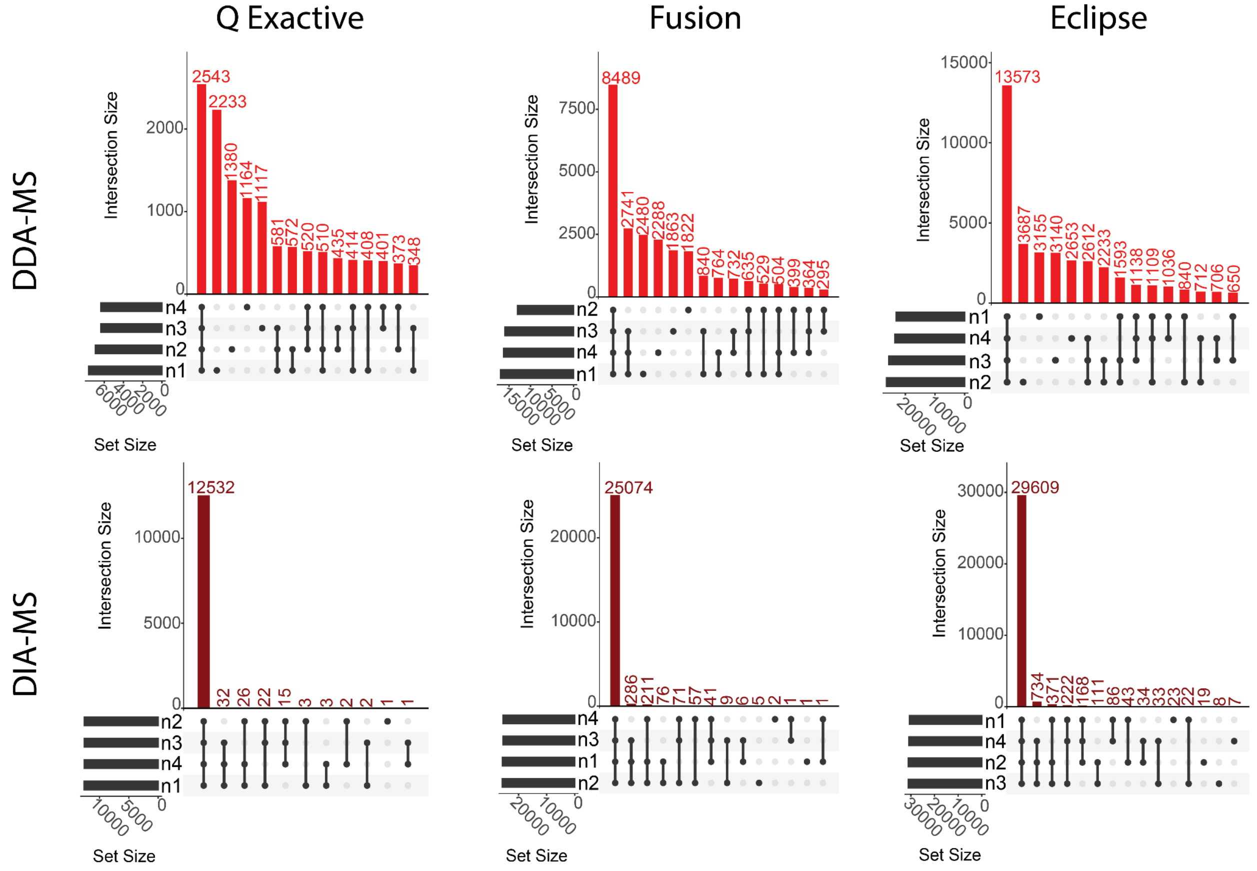

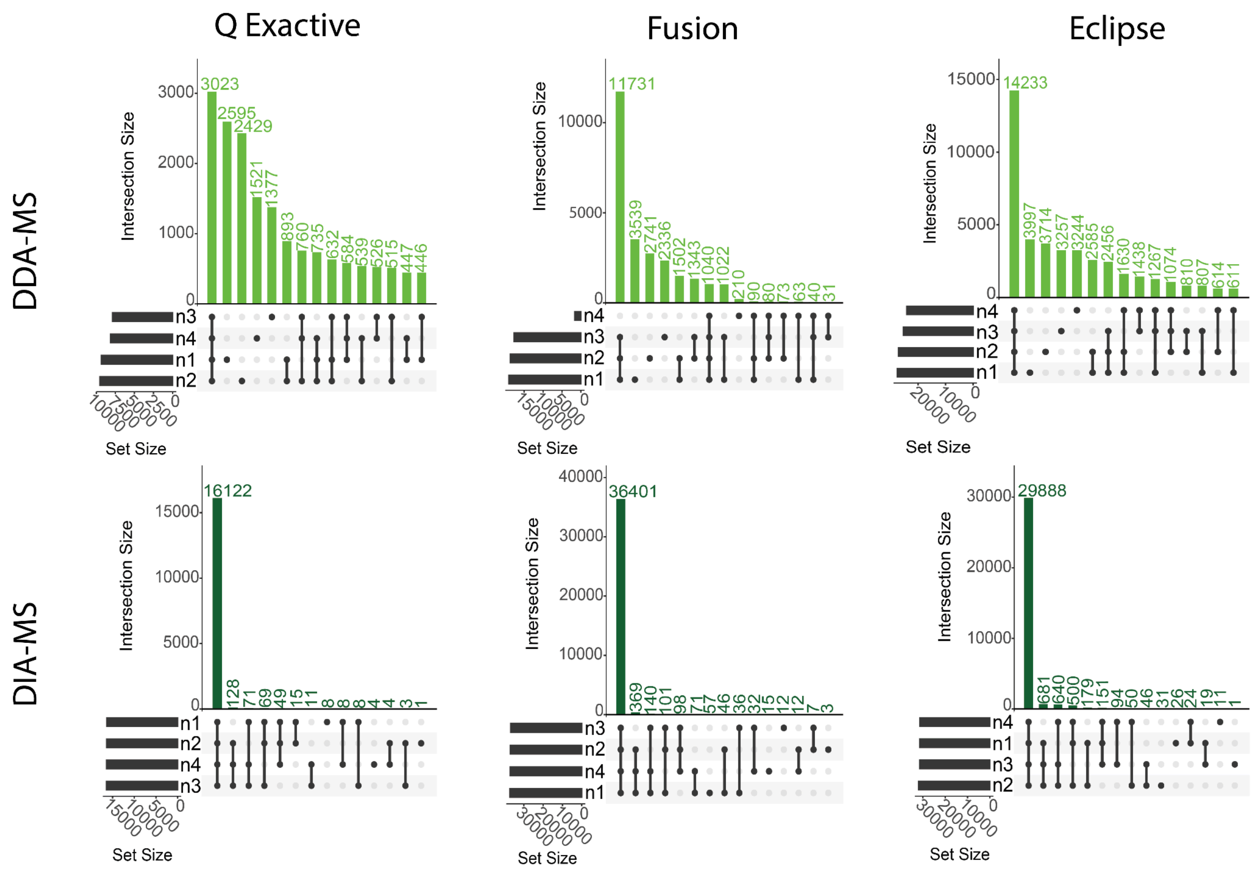
Q Exactive
Fusion
Eclipse
DDA-MS
DIA-MS
Intersection Size
Set Size
3023
2595
2429
1521
1377
893
760
735
632
584
539
526
515
447
446
11731
3539
2741
2336
1502
1343
1040
1022
210
90
80
73
63
40
31
14233
3997
3714
3257
3244
2585
2456
1630
1438
1267
1074
810
807
614
611
16122
128
71
69
49
15
11
8
8
8
4
4
3
1
36401
369
140
101
98
71
57
46
36
32
15
12
12
7
3
29888
681
640
500
179
151
94
50
46
31
26
24
19
1
n1
n2
n3
n4

Supplemental Table S2: A) Proteins and B) peptides identified via DDA-MS and DIA-MS specific to the species within the microbiome

A)

	QE Even Cell		QE Even Protein		QE Uneven		Fusion Even Cell		Fusion Even Protein		Fusion Uneven		Eclipse Even Cell		Eclipse Even Protein		Eclipse Uneven	
Species	DDA	DIA	DDA	DIA	DDA	DIA	DDA	DIA	DDA	DIA	DDA	DIA	DDA	DIA	DDA	DIA	DDA	DIA
137	1	71	17	81	3	27	2	138	31	172	7	53	5	194	35	191	8	55
259	68	106	383	508	112	163	125	188	555	804	202	326	192	245	658	782	271	337
841	144	232	301	460	109	204	251	464	461	905	234	490	502	691	748	987	361	521
AK199	124	66	141	83	41	23	197	128	230	149	100	62	388	179	375	156	134	61
Am2	28	44	96	137	19	30	53	116	162	339	45	90	113	167	250	362	57	82
ATN	111	216	149	249	225	391	218	391	258	538	408	716	413	562	413	565	617	780
BS	194	267	126	190	22	48	325	493	205	397	54	125	506	639	307	416	91	130
BXL	10	13	8	17	14	27	23	32	23	39	34	47	33	43	34	50	51	45
CRH	451	673	48	91	63	108	718	1134	87	191	121	247	1118	1605	121	211	168	261
Cup	67	125	163	272	584	908	147	272	294	613	956	1490	292	426	456	656	1284	1575
CV	159	99	105	129	44	45	271	189	173	259	81	109	475	261	282	282	109	107
DVH	3	5	5	8	22	45	6	16	9	18	44	116	6	12	10	12	88	148
ES18	1	2	0	3	1	0	2	2	2	2	0	0	2	1	2	2	0	0
F0	0	0	2	3	1	1	0	2	3	5	1	2	1	0	4	4	1	2
F2	1	1	1	1	0	0	1	1	1	1	1	1	1	1	1	1	1	0
HB2	87	158	132	218	24	51	179	360	251	529	64	192	396	602	431	672	143	263
K12	192	272	238	327	302	416	292	413	351	574	453	683	410	499	453	567	583	660
KF7	130	194	111	170	30	54	215	358	163	356	67	126	420	566	289	407	108	129
LT2	127	173	145	210	295	454	205	356	228	492	498	840	370	462	360	475	705	865
M13	0	0	0	0	0	0	1	1	0	1	0	0	0	0	0	0	0	0
Ne1	2	6	4	5	7	13	8	13	6	9	11	21	11	17	11	13	17	24
Nm1	6	3	5	5	6	14	7	8	6	9	10	24	11	15	12	21	17	22
Nu1	3	5	4	5	3	5	6	11	7	11	8	9	10	11	14	10	10	10
NV	3	10	83	161	10	23	9	16	176	357	20	75	18	37	250	371	47	88
P22	0	0	2	2	1	1	0	0	1	4	1	1	0	0	3	6	2	2
PaD	41	252	100	317	15	92	83	519	182	687	44	241	157	733	254	699	77	245
PD	150	232	140	225	97	155	252	421	249	482	203	373	433	569	372	511	289	412
Pfl	103	168	119	217	230	364	181	331	222	507	419	697	316	433	321	452	602	733
SMS	145	214	173	245	345	538	224	404	255	556	592	987	425	599	417	615	827	1068

VF	11	17	19	26	12	18	16	24	24	58	16	28	23	37	39	62	23	38

B)

	QE Equal Cell		QE Equal Protein		QE Uneven		Fusion Equal Cell		Fusion Equal Protein		Fusion Uneven		Eclipse Equal Cell		Eclipse Equal Protein		Eclipse Uneven	
Species	DDA	DIA	DDA	DIA	DDA	DIA	DDA	DIA	DDA	DIA	DDA	DIA	DDA	DIA	DDA	DIA	DDA	DIA
137	4	209	39	206	5	51	8	455	92	527	22	149	16	559	118	482	26	117
259	138	290	1470	2610	267	524	374	618	2741	4607	685	1350	597	700	3411	3829	1035	1183
841	334	641	732	1469	234	494	796	1427	1576	3485	667	1417	1469	1838	2387	2913	1095	1305
AK199	274	141	337	174	82	33	590	318	719	386	260	120	1063	416	1082	336	388	118
Am2	65	106	234	403	43	46	149	276	476	1081	143	187	316	333	780	897	187	150
ATN	223	492	315	630	472	1067	580	1066	731	1648	1233	2496	1094	1385	1143	1422	1965	2407
BS	548	1028	350	646	66	103	1249	2094	747	1610	192	321	1983	2380	1103	1315	308	307
BXL	44	25	56	24	60	33	155	55	173	69	191	60	178	69	192	65	214	51
CRH	1315	2383	209	206	235	262	2779	4434	520	494	676	663	4008	5178	623	412	853	624
Cup	141	275	380	751	1638	3461	428	708	900	2056	3694	6962	772	951	1367	1709	5546	6752
CV	396	256	261	357	97	85	847	490	526	761	237	240	1449	613	838	662	327	221
DVH	15	5	21	8	79	131	56	18	69	19	200	337	60	14	78	13	307	350
ES18	1	2	1	4	1	0	2	5	2	6	1	0	4	3	3	3	1	0
F0	1	0	3	8	1	1	2	2	10	19	2	4	1	0	14	14	3	3
F2	1	2	1	1	0	0	2	2	2	2	1	2	2	2	2	1	84	0
HB2	207	453	331	693	56	119	569	1257	790	2064	190	516	1234	1958	1417	2245	1006	651
K12	522	951	703	1327	964	1746	1079	1659	1355	2473	1868	3153	1488	1799	1784	2051	2072	2976
KF7	267	464	212	371	59	82	620	1000	454	948	188	238	1141	1318	787	862	771	213
LT2	260	441	320	551	700	1242	540	942	629	1424	1467	2919	920	1091	957	1174	1769	2674
M13	0	0	0	0	0	0	2	1	0	1	0	0	0	0	0	0	0	0
Ne1	12	7	16	7	19	15	35	15	43	10	56	37	47	20	54	13	66	32
Nm1	18	4	16	6	16	14	41	10	47	12	60	31	54	17	61	23	68	27
Nu1	14	5	15	5	16	7	44	12	48	11	45	11	53	12	63	10	80	10
NV	23	26	194	437	41	54	64	41	505	1214	101	160	81	68	715	968	122	167
P22	0	0	8	14	1	3	1	0	12	24	8	8	1	0	18	25	50	7
PaD	75	736	180	832	40	191	189	1636	401	2068	120	578	342	1948	552	1680	414	502
PD	326	598	313	607	201	357	707	1222	707	1455	541	1005	1256	1513	1089	1322	1124	1048
Pfl	243	418	290	629	569	1098	517	944	660	1554	1374	2536	892	1086	965	1196	2496	2441
SMS	360	709	449	825	1059	2127	783	1433	918	2142	2340	4524	1360	1759	1411	1830	2726	4384
VF	24	44	52	95	24	47	66	86	117	195	74	99	98	103	149	170	90	95

Supplemental Figure S2: Scatter plots of reference and measured percent abundances of species in the UNEVEN samples. Average Spearman correlation values show no significant difference to one another.

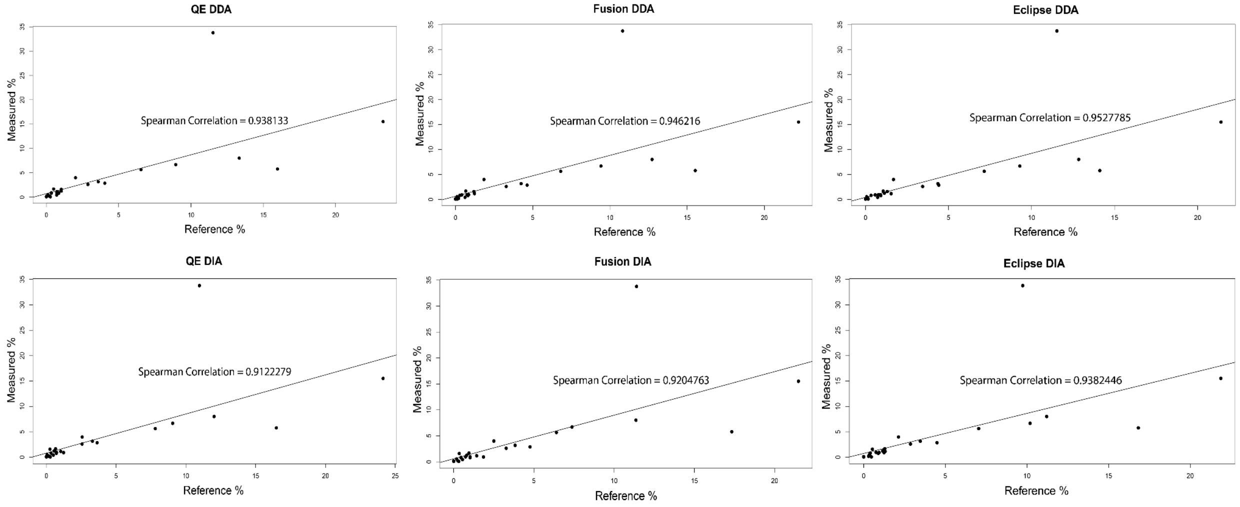

Supplemental Table S3: Fractional abundances of microbiome species in a) Equal Cell Number samples, b) Equal Protein Amount samples, and c) Uneven samples. Species in each sample type are listed from most to least abundant. DDA and DIA abundances for each species were compared via Student's t-test, with the p-value given indicating the significance of the difference between the values.

a)

ECN	Q Exactive			Fusion			Eclipse		
	DDA	DIA	p-value	DDA	DIA	p-value	DDA	DIA	p-value
CRH	0.041598	0.047943	0.002366	0.072735	0.080826	0.108138	0.10462	0.114422	0.003014
CV	0.046806	0.024359	0.000152	0.081956	0.046559	9.39E-05	0.136532	0.064443	8.13E-07
Pfl	0.023745	0.028596	0.058204	0.042851	0.056298	0.009859	0.067447	0.073617	0.191603
KF7	0.029227	0.034741	0.023494	0.052403	0.064147	0.009941	0.092613	0.101488	8.82E-05
PD	0.036185	0.046267	0.007819	0.063037	0.084049	0.000183	0.103863	0.113596	0.020628
SMS	0.04428	0.053625	0.03936	0.075828	0.101229	0.002935	0.128512	0.150339	0.003591
AK199	0.041793	0.018177	1.55E-06	0.069058	0.035114	0.000161	0.127582	0.04916	9.89E-07
HB2	0.049566	0.072065	0.001891	0.104728	0.16423	3.94E-05	0.206373	0.274897	9.47E-07
ATN	0.027246	0.040471	1.11E-06	0.053039	0.073298	0.000212	0.094917	0.105468	0.013085
BS	0.052195	0.063708	0.003601	0.09419	0.117633	2.59E-05	0.139048	0.15247	0.015094
841	0.027616	0.034763	7.65E-05	0.050853	0.069451	0.001759	0.092464	0.10339	0.000412
LT2	0.038252	0.0448	0.007797	0.065807	0.092324	0.002154	0.112033	0.119684	0.003062
K12	0.051999	0.064672	6.89E-05	0.082699	0.098346	0.00352	0.111851	0.118753	0.001998
Cup	0.015494	0.019763	0.013909	0.037826	0.042964	0.147164	0.06419	0.067273	0.238742
Am2	0.011677	0.011355	0.790402	0.024323	0.029935	0.03558	0.048194	0.043097	0.199194
PaD	0.010355	0.044138	2.07E-08	0.022771	0.091128	2.67E-09	0.037996	0.128598	4.81E-11
VF	0.009589	0.011855	0.286738	0.022838	0.016736	0.001836	0.030335	0.025976	0.031126
M13	0.027778	0	0.355918	0.166667	0.111111	0.133975	0	0	-
BXL	0.004823	0.00152	7.28E-06	0.016517	0.003742	0.000301	0.018475	0.004999	1.24E-05
F2	0.1875	0.25	0.355918	0.25	0.25	-	0.25	0.25	-
ES18	0.013158	0.026316	0.133975	0.026316	0.026316	1	0.039474	0.013158	0.002714
137	0.004428	0.114332	4.14E-11	0.010467	0.22182	2.98E-11	0.020531	0.312802	2.54E-10
NV	0.004256	0.003212	0.2914	0.014616	0.00514	0.000232	0.018712	0.011966	0.002995

259	0.032604	0.041022	0.00391	0.060275	0.072755	0.027848	0.088913	0.094814	0.000461
F0	0.002273	0	0.133975	0.007955	0.007955	1	0.005682	0	0.094133
P22	0.00463	0	0.355918	0.013889	0	0.168227	0.013889	0	0.168227
DVH	0.004226	0.001433	0.0005	0.015759	0.004585	3.97E-05	0.016547	0.003295	8.83E-05
Ne1	0.005015	0.002561	0.013133	0.014405	0.005548	0.000177	0.019206	0.007149	1.42E-05
Nm1	0.006285	0.001109	3.23E-07	0.014418	0.002957	7.45E-05	0.018762	0.00536	0.000221
Nu1	0.004993	0.001816	0.008695	0.015704	0.003994	0.000197	0.019154	0.003903	4.78E-06

b)

EPA	Q Exactive			Fusion			Eclipse		
	DDA	DIA	p-value	DDA	DIA	p-value	DDA	DIA	p-value
LT2	0.005187	0.054461	2.24E-11	0.070799	0.127658	3.27E-08	0.110931	0.12312	0.027458
PD	0.082152	0.044919	5.48E-06	0.065033	0.096177	4.39E-06	0.093631	0.101917	0.103823
BS	0.084884	0.045216	0.000186	0.065378	0.094667	6.76E-06	0.092818	0.099141	0.183962
PaD	0.030932	0.055546	1.75E-06	0.041945	0.120612	7.27E-12	0.05616	0.122631	8.71E-08
AK199	0.033737	0.022859	0.000463	0.07973	0.041036	8.74E-06	0.123933	0.043032	2.72E-05
KF7	0.03371	0.030393	0.112695	0.043886	0.063789	0.000176	0.070109	0.072889	0.577765
CV	0.038789	0.031759	0.020839	0.059571	0.063764	0.000715	0.089973	0.069623	0.003549
ATN	0.010223	0.046614	4.99E-09	0.063356	0.100966	2.8E-07	0.095808	0.105937	0.02545
SMS	0.001317	0.061465	4.28E-15	0.083668	0.139488	3.63E-08	0.127321	0.154227	0.004756
Cup	0.025099	0.042964	3.1E-06	0.061581	0.096877	1.13E-06	0.093478	0.103755	0.01986
Pfl	0.037021	0.036894	0.945394	0.052596	0.086213	0.002038	0.073191	0.076894	0.670084
BXL	0.015669	0.001988	5.27E-08	0.019089	0.004531	4.5E-07	0.020989	0.005847	1.54E-07
137	0.032609	0.130435	2.94E-06	0.061997	0.27657	2.72E-11	0.073269	0.307568	5.44E-10
259	0.000484	0.196594	9.73E-19	0.236068	0.311146	8.89E-08	0.275348	0.302632	0.012693
Am2	0.000516	0.035419	6.84E-13	0.058903	0.087548	3.89E-07	0.085935	0.09329	0.181881
K12	0.010531	0.077701	0.000618	0.097394	0.136602	2.5E-06	0.125595	0.159864	7.92E-05
HB2	0.088282	0.099703	0.477159	0.138648	0.241663	3.73E-08	0.223732	0.258908	0.002279
CRH	0.016433	0.006505	0.002568	0.028249	0.013599	1.97E-08	0.032348	0.015025	1.11E-06
841	0.022938	0.068889	2.23E-06	0.086477	0.135496	1.45E-07	0.132727	0.14777	0.013839
VF	0.089435	0.018131	0.034949	0.03016	0.040098	8.55E-07	0.043236	0.042887	0.938328
NV	0.005381	0.051558	4.23E-10	0.075169	0.114761	0.000288	0.097334	0.119017	0.033991
M13	0	0	-	0.027778	0.111111	0.024008	0	0	-
F2	0.25	0.25	-	0.25	0.25	-	0.25	0.25	-
P22	0.037037	0.032407	0.62022	0.050926	0.074074	0.094133	0.083333	0.111111	0.168227
F0	0.010227	0.013636	0.168227	0.020455	0.022727	0.5847	0.029545	0.018182	0.027811

ES18	0.009868	0.039474	0.000105	0.029605	0.026316	0.355918	0.029605	0.026316	0.355918
DVH	0.045129	0.002292	6.69E-05	0.019413	0.005158	2.36E-06	0.021848	0.003438	1.74E-06
Ne1	0.074584	0.002027	0.000257	0.017926	0.003948	2.4E-05	0.021874	0.005335	2.41E-07
Nm1	0.061738	0.001848	0.014631	0.016913	0.003235	3.67E-06	0.021257	0.007671	8.6E-06
Nu1	0.006445	0.001816	0.088498	0.017248	0.003994	4.74E-05	0.022331	0.00345	6.96E-06

c)

Uneven	Q Exactive			Fusion			Eclipse		
	DDA	DIA	p-value	DDA	DIA	p-value	DDA	DIA	p-value
LT2	0.083182	0.117674	0.0009	0.1427	0.217713	1.29E-05	0.200402	0.224196	0.049676
Cup	0.102964	0.143478	3.97E-07	0.169763	0.235494	3.39E-05	0.227708	0.248972	0.002486
SMS	0.098595	0.135035	9.65E-09	0.167963	0.247679	2.96E-05	0.234069	0.267875	0.000163
Pfl	0.047362	0.061957	3.29E-06	0.08766	0.118553	2.13E-05	0.122723	0.124766	0.236677
K12	0.079069	0.09906	0.003002	0.120895	0.162542	6.22E-06	0.154569	0.157009	0.63211
ATN	0.049709	0.073345	2.51E-05	0.093744	0.134356	3.23E-05	0.136278	0.146361	0.007081
CRH	0.012797	0.007682	0.00041	0.034576	0.017609	9.49E-05	0.03978	0.018625	1.4E-10
PD	0.025105	0.030994	0.036784	0.0556	0.074516	0.002524	0.077361	0.082252	0.385001
VF	0.011681	0.012378	0.346422	0.026848	0.019526	0.01308	0.031206	0.026151	0.071268
HB2	0.015418	0.023298	0.001473	0.046368	0.087597	2.25E-06	0.087825	0.12026	0.001514
259	0.050019	0.062984	0.001116	0.09404	0.126064	6.83E-05	0.121904	0.130418	0.170644
AK199	0.016249	0.006265	0.001151	0.045098	0.016937	2.68E-06	0.056803	0.016662	4.33E-05
CV	0.01665	0.0111	5.33E-05	0.038234	0.026764	9.34E-05	0.046929	0.026394	9.75E-06
KF7	0.009638	0.009638	1	0.026986	0.022548	0.017457	0.036624	0.023041	6.01E-05
137	0.006441	0.043478	5.62E-08	0.026973	0.085749	8.7E-08	0.025765	0.088164	3.66E-08
Am2	0.009355	0.007677	0.021707	0.029548	0.023097	0.002101	0.033484	0.021161	0.000666
DVH	0.012249	0.012894	0.520897	0.029656	0.033166	0.004196	0.044986	0.042407	0.157125
PaD	0.005923	0.016058	3.78E-07	0.017111	0.042296	2.06E-07	0.022552	0.042954	3.7E-09
841	0.021741	0.03046	1.48E-05	0.050142	0.073342	6.33E-05	0.073043	0.077982	0.024008
NV	0.007549	0.007388	0.873684	0.021924	0.024012	0.083052	0.031561	0.028188	0.094264
BS	0.009962	0.011393	0.04925	0.027977	0.029707	0.007255	0.038654	0.030959	2.47E-05
Nu1	0.005628	0.001816	1.91E-05	0.01634	0.003359	4.32E-07	0.022059	0.00354	0.000225
BXL	0.007104	0.003128	4.06E-06	0.02134	0.005437	9.62E-06	0.023913	0.005203	1.39E-08
Nm1	0.0061	0.005176	0.421936	0.02098	0.008965	2.27E-05	0.023383	0.007948	0.000282
M13	0.027778	0	0.355918	0	0	-	0	0	-

P22	0.013889	0.018519	0.355918	0.060185	0.018519	0.003326	0.041667	0.037037	0.355918
F0	0.003409	0.004545	0.355918	0.007955	0.009091	0.779559	0.010227	0.009091	0.355918
ES18	0.006579	0	0.133975	0.016447	0	0.14663	0.006579	0	0.355918
F2	0	0	-	0.125	0.25	0.133975	0.1875	0	0.024008
Ne1	0.007469	0.005442	0.000472	0.022514	0.008856	2.33E-05	0.026248	0.01035	5.92E-06

Supplemental Figure S3: Distribution of misidentified proteins matching to each entrapment genome. (A) A stacked bar chart depicting the average number of proteins identified with at least 1 unique peptide from each of the entrapment genomes added to the A1, A1I1, and A1I2 databases using the DIA method. (B) A stacked bar chart depicting the average number of proteins identified with at least 1 unique peptide from each of the entrapment genomes added to the A1, A1I1, and A1I2 databases using the DDA method

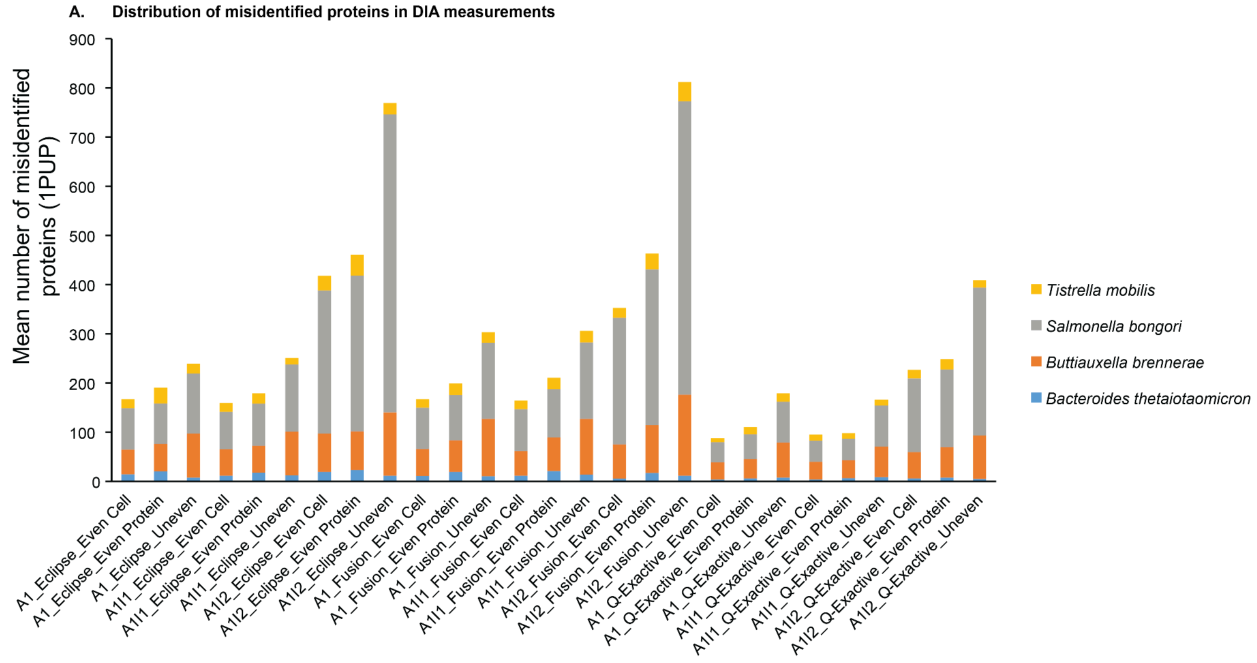

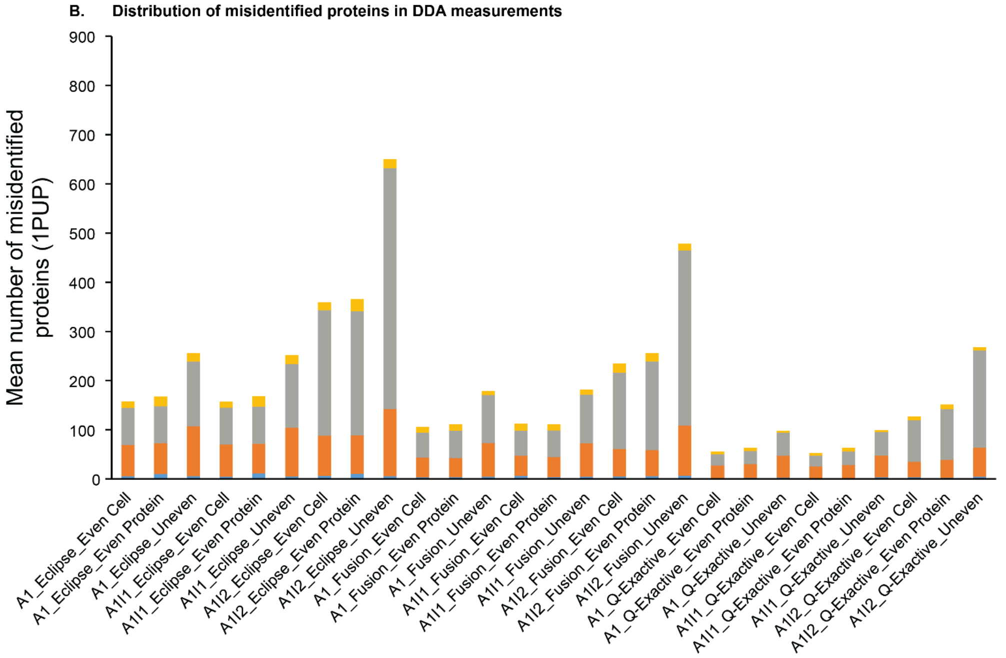

Supplement: Supplement 1 [file media-1.pdf]
