# Supplementary material for: Data-Independent Acquisition Mass Spectrometry as a Tool for Metaproteomics: Interlaboratory Comparison Using a Model Microbiome
Source: bioRxiv. 2024 Sep 22:2024.09.18.613707. Preprint. [Version 1] doi: 10.1101/2024.09.18.613707 (PMC11430069; doi:10.1101/2024.09.18.613707)

Supplemental Information for Data-Independent Acquisition Mass Spectrometry as a Tool for Metaproteomics: Cross-Laboratory Methodological Comparisons Using a Model Microbiome

Rajczewski, A. T.[1], Blakeley-Ruiz, J. A.[2], Meyer, A.[3], Vintila, S.[2], McIlvin, M.R.[4], Van Den Bossche, T.[5, 6], Searle, B.C.[7], Griffin, T.J.[1], Saito, M.[4], Kleiner, M.[2], Jagtap, P.D.[1]

[1] Department of Biochemistry, Molecular Biology, and Biophysics, University of Minnesota, Minneapolis MN USA
[2] Department of Plant and Microbial Biology, North Carolina State University, Raleigh NC USA
[3] MIT-WHOI Joint Program in Oceanography/Applied Ocean Science and Engineering, Department of Chemistry, Woods Hole Oceanographic Institution, Woods Hole MA USA, Department of Earth, Atmospheric, and Planetary Sciences, Massachusetts Institute of Technology, Cambridge MA USA
[4] Department of Marine Chemistry and Geochemistry, Woods Hole Oceanographic Institution, Woods Hole MA USA
[5] VIB-UGent Center for Medical Biotechnology, VIB,, Ghent Belgium
[6] Department of Biomolecular Medicine, Ghent University, Ghent Belgium
[7] Department of Chemistry and Biochemistry, The Ohio State University, Columbus OH USA

Supplemental Table 1: Protein sequence database components used in the proteomic analyses of composite microbiome samples

**Label**	**Species**	Source of protein sequences	Original Database	Incomplete 1	Incomplete 2	Added1	Added Incomplete1	Added Incomplete2
NV	*Nitrososphaera viennensis*	GCA_000698785.1	Y	Y	Y	Y	Y	Y
841	*Rhizobium leguminosarum bv. viciae* 3841	UP000006575	Y	N	N	Y	N	N
PaD	*Paracoccus denitrificans*	Used RAST. Available in the protein sequence database on PRIDE.	Y	Y	Y	Y	Y	Y
Cup	*Cupriavidus metallidurans*	UP000002429	Y	Y	Y	Y	Y	Y
CV	*Chromobacterium violaceum*	Used RAST. Available in the protein sequence database on PRIDE.	Y	Y	Y	Y	Y	Y
Nu1	*Nitrosomonas ureae*	UP000056699	Y	Y	Y	Y	Y	Y
DVH	*Desulfovibrio vulgaris*	UP000002194	Y	Y	Y	Y	Y	Y
K12	*Escherichia coli*	UP000000625	Y	Y	Y	Y	Y	Y
PD	*Pseudomonas denitrificans*	UP000012082	Y	N	N	Y	N	N
KF7	*Pseudomonas pseudoalcaligenes*	GCA_000262065.3	Y	Y	N	Y	Y	N
Pfl	*Pseudomonas fluorescens*	2617270901 (IMG)	Y	N	N	Y	N	N
HB2	*Thermus Thermophilus*	UP000000592	Y	Y	Y	Y	Y	Y
CRH	*Chlamydomonas reinhardtii*	GCF_000002595.1	Y	Y	Y	Y	Y	Y
LT2	*Salmonella enterica Typhimurium* (3 strains combined)	UP000001014	Y	Y	N	Y	Y	N

ATN	*Agrobacterium tumefaciens*	UP00000813	Y	Y	Y	Y	Y	Y
VF	*Rhizobium leguminosarum bv. viciae* VF39	GCA_000427765.1	Y	Y	N	Y	Y	N
AK199	*Roseobacter sp.* AK199	Used RAST. Available in the protein sequence database on PRIDE.	Y	Y	Y	Y	Y	Y
BXL	*Burkholderia xenovorans*	UP000001817	Y	Y	Y	Y	Y	Y
Ne1	*Nitrosomonas europaeae*	UP000001416	Y	Y	Y	Y	Y	Y
Nm1	*Nitrosospira multiformis*	UP000002718	Y	Y	Y	Y	Y	Y
Am2	*Alteromonas macleodii*	UP000006296	Y	Y	Y	Y	Y	Y
SMS	*Stenotrophomonas maltophilia*	GCF_000613205.1	Y	Y	Y	Y	Y	Y
BS	*Bacillus subtilis*	UP000001570	Y	Y	Y	Y	Y	Y
137	*Staphylococcus aureus* ATCC 13709	Used RAST.. Available in the protein sequence database on PRIDE	Y	Y	Y	Y	Y	Y
259	*Staphylococcus aureus* ATCC 25923	GCA_000756205.1	Y	Y	Y	Y	Y	Y
ES18	Phage ES18	UP000000970	Y	Y	Y	Y	Y	Y
F0	Phage F0	UP000009070	Y	Y	Y	Y	Y	Y
F2	Phage F2	UP000002127	Y	Y	Y	Y	Y	Y
M13	Phage M13	UP000002111	Y	Y	Y	Y	Y	Y
P22	Phage P22	UP000007960	Y	Y	Y	Y	Y	Y
BT	*Bacteroides thetaiotaomicron*	UP00000141	N	N	N	Y	Y	Y
BBE	*Buttiauxella brennerae*	UP00007841	N	N	N	Y	Y	Y
SBI	*Salmonella bongori*	UP00027208	N	N	N	Y	Y	Y
TMO	*Tistrella mobilis*	UP00000525	N	N	N	Y	Y	Y

Supplemental Figure 1: UpSet plots of peptide reproducibility of Equal Cell Number (red) and Equal Protein Amount (green) communities across three mass spectrometers (QExactive, Fusion, and Eclipse) under DDA- and DIA-MS analysis. The variables n1 through n4 represent MS runs of the four replicates.

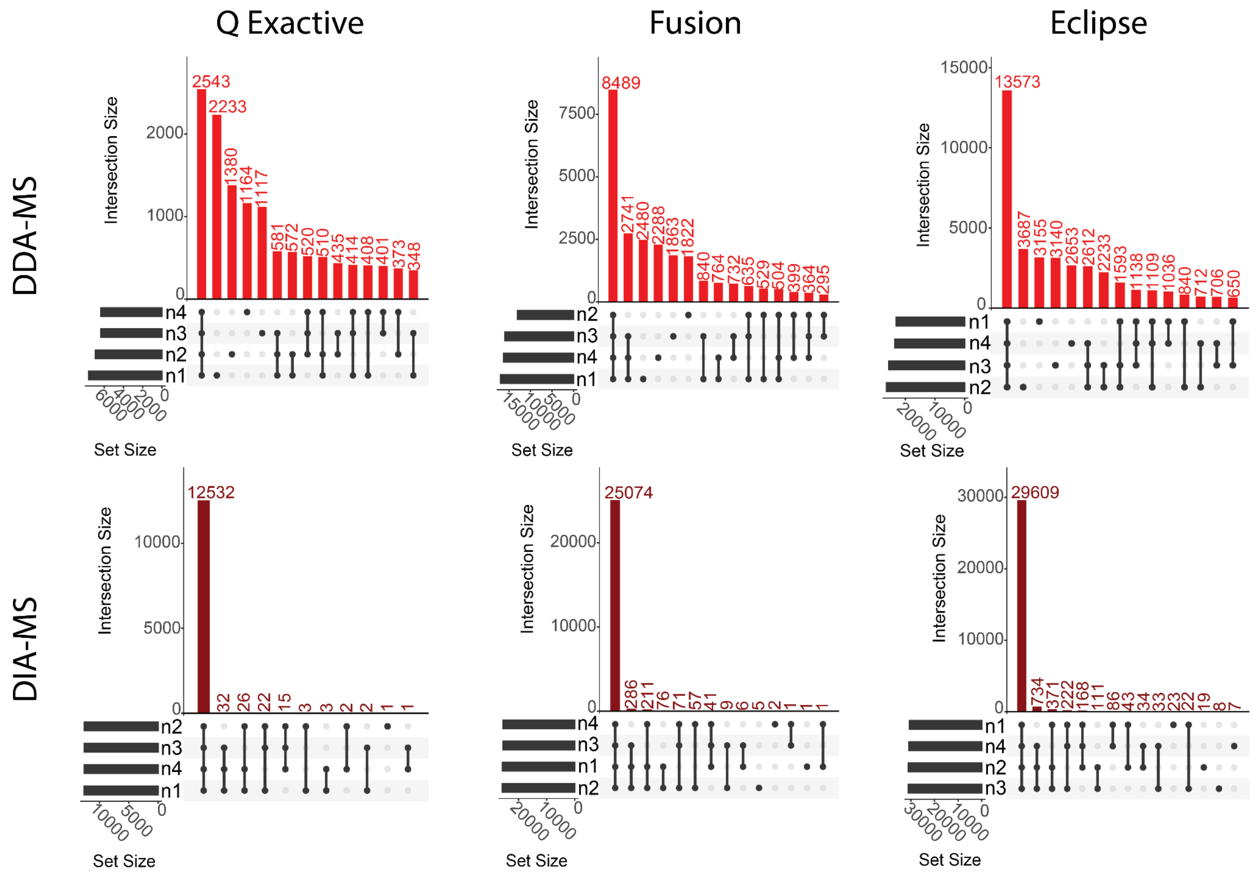

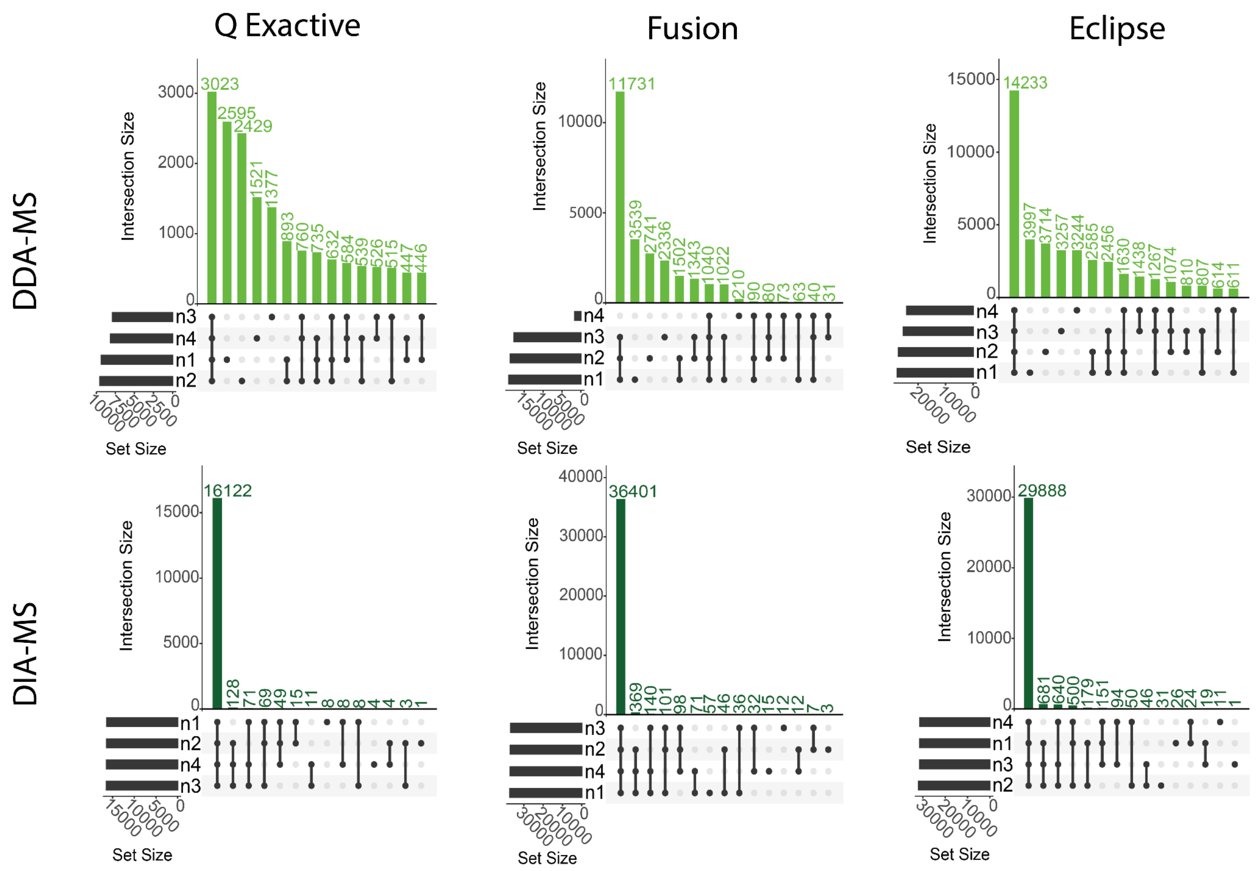

Q Exactive
Fusion
Eclipse
DDA-MS
DIA-MS
Intersection Size
Set Size

Supplemental Figure 2: Scatter plots of reference and measured percent abundances of species in the UNEVEN samples. Average Spearman correlation values show no significant difference to one another.

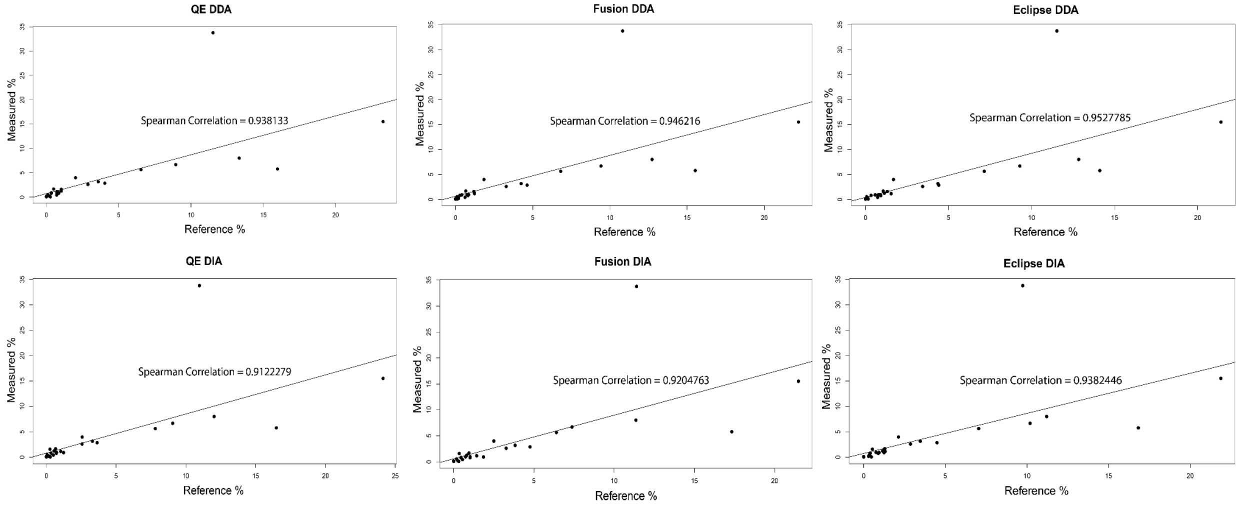

Supplemental Table 2: Fractional abundances of microbiome species in a) Equal Cell Number samples, b) Equal Protein Amount samples, and c) Uneven samples. Species in each sample type are listed from most to least abundant. DDA and DIA abundances for each species were compared via Student's t-test, with the p-value given indicating the significance of the difference between the values.

a)

ECN	Q Exactive			Fusion			Eclipse		
	DDA	DIA	p-value	DDA	DIA	p-value	DDA	DIA	p-value
CRH	0.041598	0.047943	0.002366	0.072735	0.080826	0.108138	0.10462	0.114422	0.003014
CV	0.046806	0.024359	0.000152	0.081956	0.046559	9.39E-05	0.136532	0.064443	8.13E-07
Pfl	0.023745	0.028596	0.058204	0.042851	0.056298	0.009859	0.067447	0.073617	0.191603
KF7	0.029227	0.034741	0.023494	0.052403	0.064147	0.009941	0.092613	0.101488	8.82E-05
PD	0.036185	0.046267	0.007819	0.063037	0.084049	0.000183	0.103863	0.113596	0.020628
SMS	0.04428	0.053625	0.03936	0.075828	0.101229	0.002935	0.128512	0.150339	0.003591
AK199	0.041793	0.018177	1.55E-06	0.069058	0.035114	0.000161	0.127582	0.04916	9.89E-07
HB2	0.049566	0.072065	0.001891	0.104728	0.16423	3.94E-05	0.206373	0.274897	9.47E-07
ATN	0.027246	0.040471	1.11E-06	0.053039	0.073298	0.000212	0.094917	0.105468	0.013085
BS	0.052195	0.063708	0.003601	0.09419	0.117633	2.59E-05	0.139048	0.15247	0.015094
841	0.027616	0.034763	7.65E-05	0.050853	0.069451	0.001759	0.092464	0.10339	0.000412
LT2	0.038252	0.0448	0.007797	0.065807	0.092324	0.002154	0.112033	0.119684	0.003062
K12	0.051999	0.064672	6.89E-05	0.082699	0.098346	0.00352	0.111851	0.118753	0.001998
Cup	0.015494	0.019763	0.013909	0.037826	0.042964	0.147164	0.06419	0.067273	0.238742
Am2	0.011677	0.011355	0.790402	0.024323	0.029935	0.03558	0.048194	0.043097	0.199194
PaD	0.010355	0.044138	2.07E-08	0.022771	0.091128	2.67E-09	0.037996	0.128598	4.81E-11
VF	0.009589	0.011855	0.286738	0.022838	0.016736	0.001836	0.030335	0.025976	0.031126
M13	0.027778	0	0.355918	0.166667	0.111111	0.133975	0	0	-
BXL	0.004823	0.00152	7.28E-06	0.016517	0.003742	0.000301	0.018475	0.004999	1.24E-05
F2	0.1875	0.25	0.355918	0.25	0.25	-	0.25	0.25	-
ES18	0.013158	0.026316	0.133975	0.026316	0.026316	1	0.039474	0.013158	0.002714
137	0.004428	0.114332	4.14E-11	0.010467	0.22182	2.98E-11	0.020531	0.312802	2.54E-10
NV	0.004256	0.003212	0.2914	0.014616	0.00514	0.000232	0.018712	0.011966	0.002995

259	0.032604	0.041022	0.00391	0.060275	0.072755	0.027848	0.088913	0.094814	0.000461
F0	0.002273	0	0.133975	0.007955	0.007955	1	0.005682	0	0.094133
P22	0.00463	0	0.355918	0.013889	0	0.168227	0.013889	0	0.168227
DVH	0.004226	0.001433	0.0005	0.015759	0.004585	3.97E-05	0.016547	0.003295	8.83E-05
Ne1	0.005015	0.002561	0.013133	0.014405	0.005548	0.000177	0.019206	0.007149	1.42E-05
Nm1	0.006285	0.001109	3.23E-07	0.014418	0.002957	7.45E-05	0.018762	0.00536	0.000221
Nu1	0.004993	0.001816	0.008695	0.015704	0.003994	0.000197	0.019154	0.003903	4.78E-06

b)

EPA	Q Exactive			Fusion			Eclipse		
	DDA	DIA	p-value	DDA	DIA	p-value	DDA	DIA	p-value
LT2	0.005187	0.054461	2.24E-11	0.070799	0.127658	3.27E-08	0.110931	0.12312	0.027458
PD	0.082152	0.044919	5.48E-06	0.065033	0.096177	4.39E-06	0.093631	0.101917	0.103823
BS	0.084884	0.045216	0.000186	0.065378	0.094667	6.76E-06	0.092818	0.099141	0.183962
PaD	0.030932	0.055546	1.75E-06	0.041945	0.120612	7.27E-12	0.05616	0.122631	8.71E-08
AK199	0.033737	0.022859	0.000463	0.07973	0.041036	8.74E-06	0.123933	0.043032	2.72E-05
KF7	0.03371	0.030393	0.112695	0.043886	0.063789	0.000176	0.070109	0.072889	0.577765
CV	0.038789	0.031759	0.020839	0.059571	0.063764	0.000715	0.089973	0.069623	0.003549
ATN	0.010223	0.046614	4.99E-09	0.063356	0.100966	2.8E-07	0.095808	0.105937	0.02545
SMS	0.001317	0.061465	4.28E-15	0.083668	0.139488	3.63E-08	0.127321	0.154227	0.004756
Cup	0.025099	0.042964	3.1E-06	0.061581	0.096877	1.13E-06	0.093478	0.103755	0.01986
Pfl	0.037021	0.036894	0.945394	0.052596	0.086213	0.002038	0.073191	0.076894	0.670084
BXL	0.015669	0.001988	5.27E-08	0.019089	0.004531	4.5E-07	0.020989	0.005847	1.54E-07
137	0.032609	0.130435	2.94E-06	0.061997	0.27657	2.72E-11	0.073269	0.307568	5.44E-10
259	0.000484	0.196594	9.73E-19	0.236068	0.311146	8.89E-08	0.275348	0.302632	0.012693
Am2	0.000516	0.035419	6.84E-13	0.058903	0.087548	3.89E-07	0.085935	0.09329	0.181881
K12	0.010531	0.077701	0.000618	0.097394	0.136602	2.5E-06	0.125595	0.159864	7.92E-05
HB2	0.088282	0.099703	0.477159	0.138648	0.241663	3.73E-08	0.223732	0.258908	0.002279
CRH	0.016433	0.006505	0.002568	0.028249	0.013599	1.97E-08	0.032348	0.015025	1.11E-06
841	0.022938	0.068889	2.23E-06	0.086477	0.135496	1.45E-07	0.132727	0.14777	0.013839
VF	0.089435	0.018131	0.034949	0.03016	0.040098	8.55E-07	0.043236	0.042887	0.938328
NV	0.005381	0.051558	4.23E-10	0.075169	0.114761	0.000288	0.097334	0.119017	0.033991
M13	0	0	-	0.027778	0.111111	0.024008	0	0	-
F2	0.25	0.25	-	0.25	0.25	-	0.25	0.25	-
P22	0.037037	0.032407	0.62022	0.050926	0.074074	0.094133	0.083333	0.111111	0.168227
F0	0.010227	0.013636	0.168227	0.020455	0.022727	0.5847	0.029545	0.018182	0.027811

ES18	0.009868	0.039474	0.000105	0.029605	0.026316	0.355918	0.029605	0.026316	0.355918
DVH	0.045129	0.002292	6.69E-05	0.019413	0.005158	2.36E-06	0.021848	0.003438	1.74E-06
Ne1	0.074584	0.002027	0.000257	0.017926	0.003948	2.4E-05	0.021874	0.005335	2.41E-07
Nm1	0.061738	0.001848	0.014631	0.016913	0.003235	3.67E-06	0.021257	0.007671	8.6E-06
Nu1	0.006445	0.001816	0.088498	0.017248	0.003994	4.74E-05	0.022331	0.00345	6.96E-06

c)

Uneven	Q Exactive			Fusion			Eclipse		
	DDA	DIA	p-value	DDA	DIA	p-value	DDA	DIA	p-value
LT2	0.083182	0.117674	0.0009	0.1427	0.217713	1.29E-05	0.200402	0.224196	0.049676
Cup	0.102964	0.143478	3.97E-07	0.169763	0.235494	3.39E-05	0.227708	0.248972	0.002486
SMS	0.098595	0.135035	9.65E-09	0.167963	0.247679	2.96E-05	0.234069	0.267875	0.000163
Pfl	0.047362	0.061957	3.29E-06	0.08766	0.118553	2.13E-05	0.122723	0.124766	0.236677
K12	0.079069	0.09906	0.003002	0.120895	0.162542	6.22E-06	0.154569	0.157009	0.63211
ATN	0.049709	0.073345	2.51E-05	0.093744	0.134356	3.23E-05	0.136278	0.146361	0.007081
CRH	0.012797	0.007682	0.00041	0.034576	0.017609	9.49E-05	0.03978	0.018625	1.4E-10
PD	0.025105	0.030994	0.036784	0.0556	0.074516	0.002524	0.077361	0.082252	0.385001
VF	0.011681	0.012378	0.346422	0.026848	0.019526	0.01308	0.031206	0.026151	0.071268
HB2	0.015418	0.023298	0.001473	0.046368	0.087597	2.25E-06	0.087825	0.12026	0.001514
259	0.050019	0.062984	0.001116	0.09404	0.126064	6.83E-05	0.121904	0.130418	0.170644
AK199	0.016249	0.006265	0.001151	0.045098	0.016937	2.68E-06	0.056803	0.016662	4.33E-05
CV	0.01665	0.0111	5.33E-05	0.038234	0.026764	9.34E-05	0.046929	0.026394	9.75E-06
KF7	0.009638	0.009638	1	0.026986	0.022548	0.017457	0.036624	0.023041	6.01E-05
137	0.006441	0.043478	5.62E-08	0.026973	0.085749	8.7E-08	0.025765	0.088164	3.66E-08
Am2	0.009355	0.007677	0.021707	0.029548	0.023097	0.002101	0.033484	0.021161	0.000666
DVH	0.012249	0.012894	0.520897	0.029656	0.033166	0.004196	0.044986	0.042407	0.157125
PaD	0.005923	0.016058	3.78E-07	0.017111	0.042296	2.06E-07	0.022552	0.042954	3.7E-09
841	0.021741	0.03046	1.48E-05	0.050142	0.073342	6.33E-05	0.073043	0.077982	0.024008
NV	0.007549	0.007388	0.873684	0.021924	0.024012	0.083052	0.031561	0.028188	0.094264
BS	0.009962	0.011393	0.04925	0.027977	0.029707	0.007255	0.038654	0.030959	2.47E-05
Nu1	0.005628	0.001816	1.91E-05	0.01634	0.003359	4.32E-07	0.022059	0.00354	0.000225
BXL	0.007104	0.003128	4.06E-06	0.02134	0.005437	9.62E-06	0.023913	0.005203	1.39E-08
Nm1	0.0061	0.005176	0.421936	0.02098	0.008965	2.27E-05	0.023383	0.007948	0.000282
M13	0.027778	0	0.355918	0	0	-	0	0	-

P22	0.013889	0.018519	0.355918	0.060185	0.018519	0.003326	0.041667	0.037037	0.355918
F0	0.003409	0.004545	0.355918	0.007955	0.009091	0.779559	0.010227	0.009091	0.355918
ES18	0.006579	0	0.133975	0.016447	0	0.14663	0.006579	0	0.355918
F2	0	0	-	0.125	0.25	0.133975	0.1875	0	0.024008
Ne1	0.007469	0.005442	0.000472	0.022514	0.008856	2.33E-05	0.026248	0.01035	5.92E-06

Supplemental Figure 3: Distribution of misidentified proteins matching to each entrapment genome. (A) A stacked bar chart depicting the average number of proteins identified with at least 1 unique peptide from each of the entrapment genomes added to the A1, A1I1, and A1I2 databases using the DIA method. (B) A stacked bar chart depicting the average number of proteins identified with at least 1 unique peptide from each of the entrapment genomes added to the A1, A1I1, and A1I2 databases using the DDA method

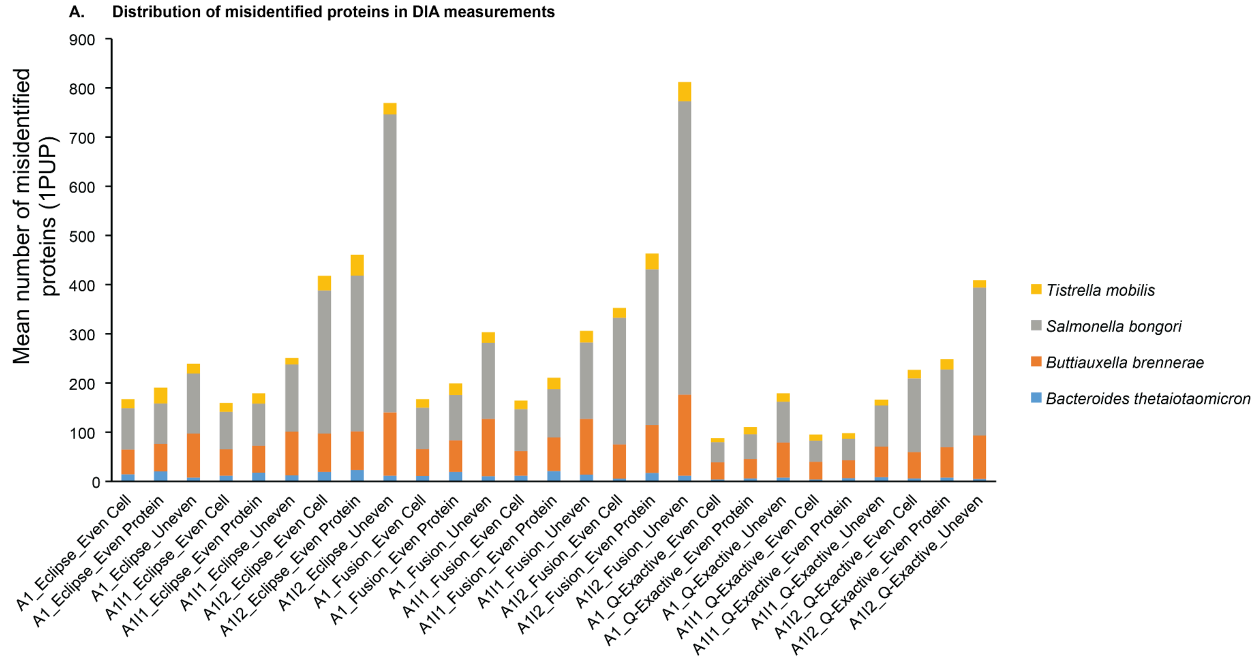

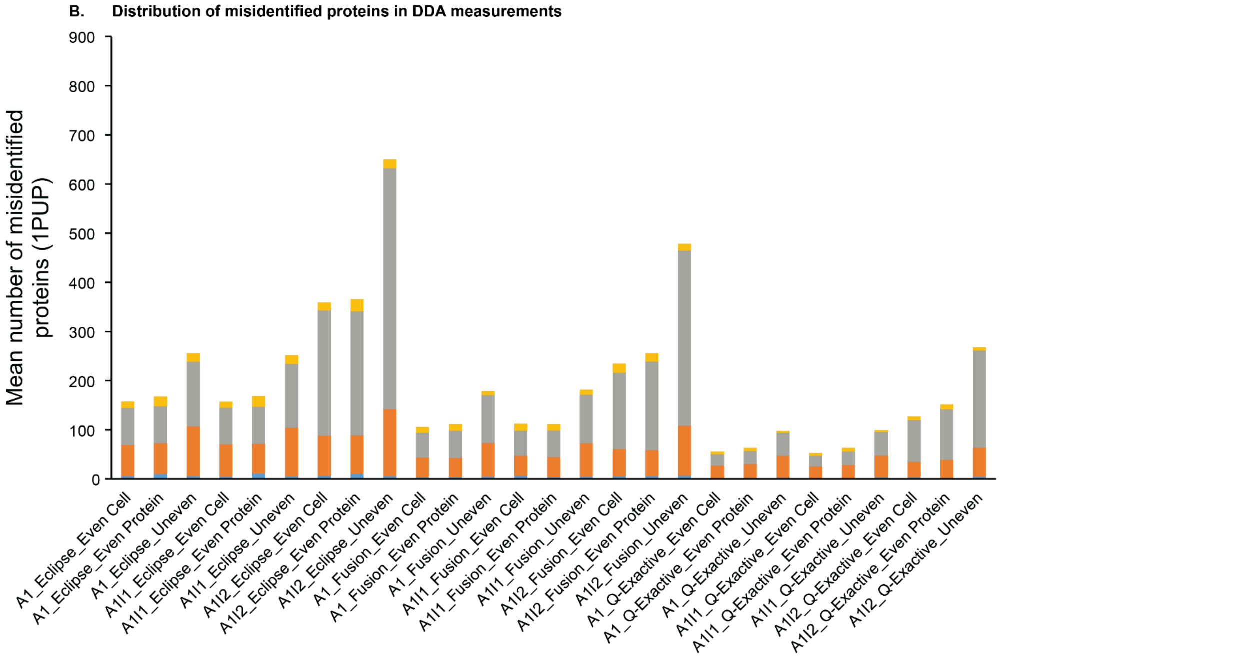

Supplement: Supplementary file 1 [file media-1.pdf]
